# Editorial: Research Model Innovations in Advancing Neonatal Care

**DOI:** 10.3389/fped.2021.711409

**Published:** 2021-06-16

**Authors:** Fook-Choe Cheah, Geok Chin Tan, Yuan Shi

**Affiliations:** ^1^Department of Paediatrics, Faculty of Medicine, Universiti Kebangsaan Malaysia Medical Centre, Kuala Lumpur, Malaysia; ^2^Department of Pathology, Faculty of Medicine, Universiti Kebangsaan Malaysia Medical Centre, Kuala Lumpur, Malaysia; ^3^Department of Neonatology, Children's Hospital of Chongqing Medical University, Chongqing, China

**Keywords:** animal models, bronchopulmonary dysplasia, developmental, hypoxic-ischemic encephalopathy, necrotizing enterocolitis, preterm, sex differences, stem cell therapy

## Introduction

Neonatology is a rapidly evolving field. Enormous progress in clinical research and translational studies bring us closer to the understanding and embracing of evidence-based practice in this specialty. While clinical trials bring research ideas of key problems to the test, often to introduce newer therapies, accurate models of diseases provide the fundamental understanding and help to answer or query why we do what we do in practice, thus critical in driving changes to improve neonatal care. In this regard, animal models and innovation prototypes, to be relevant substitutes of patients, are first rigorously studied in pre-clinical trials that simulate real clinical conditions. This special edition aims to highlight original research and innovation in models of pre-clinical studies or prototypes worthy of future clinical testing and application.

## Bronchopulmonary Dysplasia

Bronchopulmonary dysplasia (BPD) is one of the most common complications of the very premature infant (1). With the rapid development of perinatal care and medical support technology, the survival rate of these infants has significantly improved. This inadvertently leads to also an increase in the rate of a new form of chronic lung disease of prematurity, the “new” BPD. The new BPD is characterized by distal lung tissue disruption that may be initiated by intrauterine inflammation or infection and modified by extrauterine postnatal factors during the critical period of alveolar development, leading to growth arrest and simplification of these alveoli (2). In order to have a better understanding of the etiology and pathophysiology of this new form of BPD, researchers need reliable preterm animal models to study these intrauterine and early perinatal factors that may be preventable or treatable. In the article by Zhang, Chu et al., a premature rat model showed poorer growth with hyperoxia exposure and changes suggestive of alveolar disruption of the new BPD with significantly reduced radial count of the alveoli, which also appeared hyperinflated. The study by Ruan et al. displayed the same growth impairment, dismal overall physical condition, and alveolar simplification from hyperoxia. While many mouse and rat models are studied at term-born, albeit at the saccular stage of lung development, equivalent to that of the human preterm infant, these animal pups could breathe spontaneously and do not require oxygen supplementation (3). The model by Zhang, Chu et al. is interesting and simulates if additional oxygen could injure the preterm immature lung at an earlier canalicular phase and interfere with growth and their overall physical condition.

Following on the role of intrauterine exposure to infection that could be an antecedent factor in the new BPD development, Cheah et al. studied the effects of low dose intra-amniotic *Gardnerella vaginalis* (GV) injection into a pregnant rabbit model. GV is one of the most common bacteria causing chorioamnionitis in prematurity (4). The major effects were rather similar to that in the model of hyperoxia by Zhang, Chu et al. in terms of growth. The fetal pups were born with significantly lower weight and the number of alveoli was also decreased, although this was not statistically significant but the alveolar septum hypertrophied after intrauterine exposure to GV. It is to be determined if the septal thickening was the result of inflammation, followed by fibromuscular and vascular remodeling that may disrupt oxygenation and gas exchange, as the hallmark changes in the BPD lung. The concept of a “two-hit hypothesis” with antenatal and postnatal factors causing BPD may be explored in the future perhaps with the transgenic mice model reported by Cheah et al.  The GM-CSF is a pro-inflammatory cytokine that is induced and elevated by infection. Using this model, the group showed a significant influx of macrophages into the lung interstitium with increased GM-CSF. It will be interesting to study these animals exposed postnatally to hyperoxia and effects on the transition from the inflammatory to the remodeling processes that may result in the development of interstitial or septal fibrosis (5). Of note, despite being different animal models, the hyperoxia and intrauterine infection exposure resulted similarly in growth restriction. Indeed, growth restriction is a potent risk factor for BPD as the associated increase in sFlt-1 (soluble FMS-like tyrosine kinase-1) (6) has a negative impact on growth factors such as VEGF, a mediator that regulates vascular growth closely linked to the alveolarization process (7). As shown by Ruan et al., the effects of oxygen toxicity resulted in increased pulmonary vascular permeability, likely from endothelial damage, and reduced Twist1 that may potentially modify VEGF as well.

It is widely appreciated that BPD is a complex disease of multi-factorial causes and appears inevitable at least in the extremely preterm population but therapy to ameliorate this condition may be in the horizon. Since the first successful cultivation of mice embryonic stem cells in 1981 (8) by Sir Martin J. Evans who eventually won the 2007 Nobel Prize in Physiology or Medicine, stem cells have been widely investigated and regarded as a promising treatment modality of various diseases. In this Research Topic, there are three reviews on the potential use of stem cells in neonatal diseases. Chia et al. described the therapeutic potential of placental stem cells due to its immunomodulating effects on BPD. Based on the current pre-clinical and clinical studies, human amniotic epithelial cells and umbilical cord mesenchymal stem cells are of the greatest potential as therapeutic agents. Liau et al. discussed the current application of mesenchymal stromal cells (MSC) in treating neonatal diseases, discussing the mechanism of action of MSCs through paracrine effects, direct cell contact, and cell fusion. They concluded that MSC therapy while promising, raised concerns in using stem cells with aspects on safety, the risk of transmitting infectious agents, and the heterogeneity in the source of stem cells possessing variable characteristics and differentiating properties. The increasing potential of extracellular vesicles (EVs) such as exosomes is highlighted in these reviews as an emerging and novel therapeutic strategy that may eliminate the worries associated with cellular products.

## Gastrointestinal Issues

**Figure 1:**
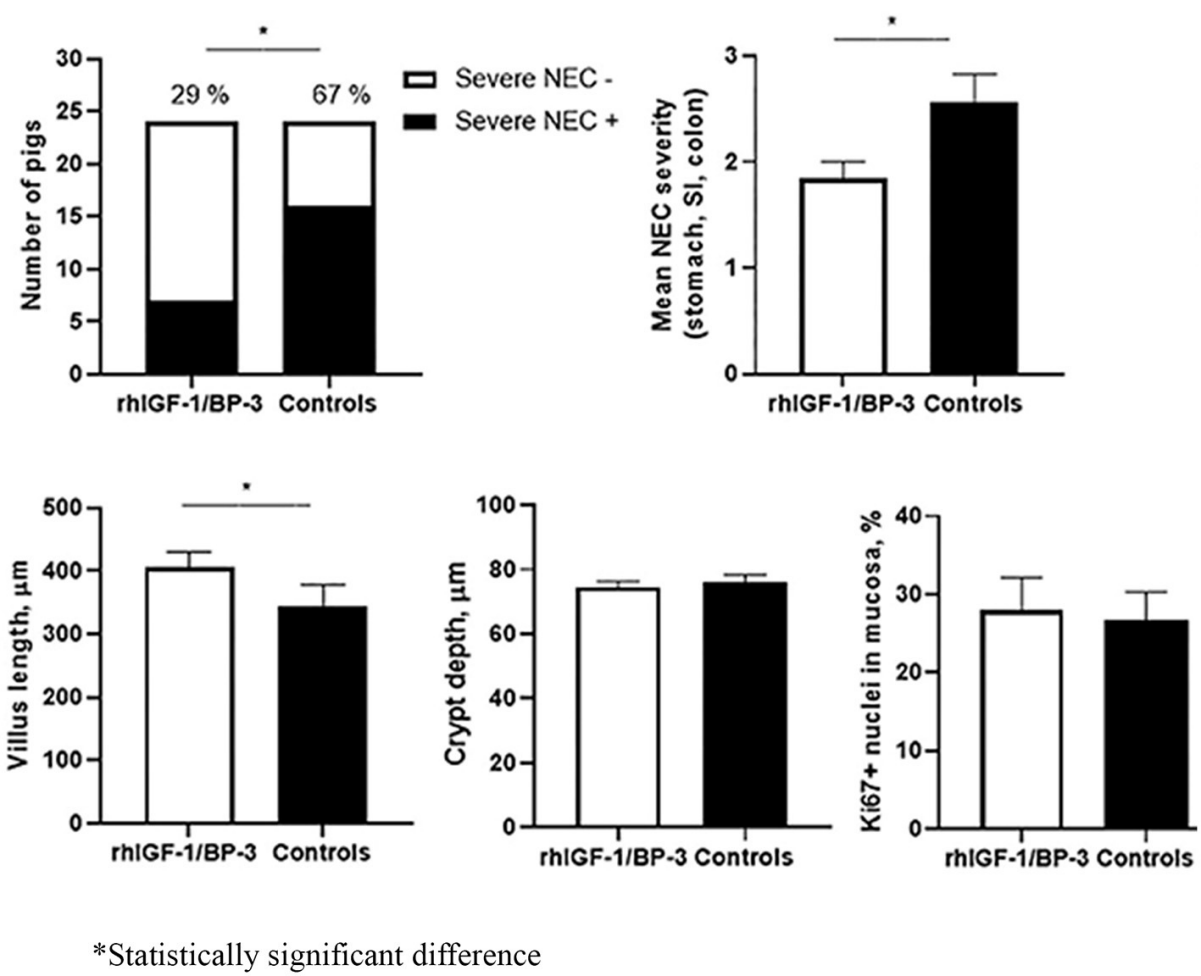
Supplemental insulin-like growth factor-1 and necrotizing enterocolitis in preterm pigs; Holgersen et al.

The other “nemesis” that neonatologists face is necrotizing enterocolitis (NEC), occurring more commonly in the very low birth weight preterm infants. Unlike BPD, this gastrointestinal complication occurs earlier, can be acute in onset and progresses unpredictably with a mortality rate that ranges from 20% to above 50% (9, 10). Once NEC sets in, supportive care is the mainstay of treatment and close observation for signs of deterioration and to decide if surgery is required. Holgersen et al. (Figure 1) showed in a study of NEC-sensitive piglets that the use of insulin-like growth factor-1 increased intestinal villus length and may be disease modifying, as it was associated with less severe NEC. Furthermore, Villamor-Martinez et al. performed a meta-analysis that concluded, both stem cells and stem cell exosomes reduced all grades of NEC in rodent-exclusive experimental models. These results are promising for future human trials but concerns raised were the lack of robustness in the animal models used, a paucity of clinician involvement and the heterogeneity in the study designs.

The two interventions proven to reduce the risk of NEC are the use of human milk as enteral feeds and probiotics (11). The availability of mother's own milk to infants born preterm are generally insufficient especially during the initial postnatal period and donor human milk (DHM) may be sourced as alternative for enteral feeding. In the article by Cayer et al., the performance of the TEMPO® system to test for human milk bacteriological contamination appears to be reliable with a quick turn-around time for the DHM to be made available for safe consumption by the recipient infants. The use of probiotics, although a standard care of practice to prevent NEC especially among the extremely preterm in some neonatal units, is an area still divided among practitioners as to the optimal timing, dose and strains to be used. Incidentally, the gut microbiome may also affect the health of other organs (12). Zhang, Ran et al. showed that acetate therapy was associated with an abundance of the microbiota that protects the lung from BPD. The mice exposed to hyperoxia but given acetate showed reduction in NLRP3-inflammasome expression and attenuated lung morphological changes of BPD. This mechanism may underlie the observation in a clinical study that showed acetate in parenteral nutrition was associated with a lower rate of BPD (13).

Intriguingly, another preventive measure to be explored that may reduce the incidence of NEC is co-bedding. Brunse et al. showed that co-bedding with skin-to-skin contact of piglet littermates improved gut function and reduced NEC incidence, independent of cortisol effects, which given as hydrocortisone to stabilize blood pressure, predisposed to NEC. Co-bedding of twins has been practiced in some neonatal intensive care units (NICUs) to simulate the intrauterine couple environment but studies have not shown any positive benefits (14). Conversely, negative implications include potential higher risks of medication errors and cross-infections. Nevertheless, the study by Brunse et al. raised hypothetically whether skin-to-skin contact, an integral feature in kangaroo mother care, gives rise to the various positive effects reported in the preterm infant (15, 16).

The other gastrointestinal-related issues faced by premature infants are gut dysmotility and maturation of the sucking-swallowing processes. These often pose as oral feeding difficulties or challenges when the infant is in the NICU and even after hospital discharge. The article by Kappel et al. provide some insights into how gastrointestinal transit time may vary based on the gestational age, postnatal age, and also the type of feeds. These could help clinicians to approach feeding intolerance occurring in their patients with the appropriate investigation and remedial plans. The review by Lau offers strategies in individualizing care of infants born preterm with different phases of maturational defects in their oral feeding skills.

## Brain Developmental Disorders and Injuries

Brain developmental disorders and injuries to the newborn infant are often devastating and result in significant long-term morbidity such as cerebral palsy. Infants with hypoxic-ischemic encephalopathy (HIE) are currently resuscitated with air and offered therapeutic hypothermia to mitigate the injurious processes in the brain. The review by Liau et al. discussed adjunct therapy with MSC that potentially attenuate the extent of the brain injury. Using the oxygen-glucose deprivation rat model, Shi et al. showed a potential target for further neuroprotection by activating Sirt-1 with the antioxidant such as resveratrol. Another innovative approach that may be explored in the future is the use of hypobaric intervention in stabilization of the asphyxiated infant with early onset and difficult to control seizures. Xie et al. showed in their model that hypobaria, simulating high-altitude environment, increases seizure threshold that may potentially reduce neuronal discharge activity and apoptosis. It may sound counter-intuitive that HIE cases are managed this way, but indeed hypoxic/ischemic pre-conditioning treatment appeared to be protective to the injured neonatal brain (17).

Studying and monitoring the oxygenation status at the cellular level in such critical cases may be complemented by the non-invasive Cellular Oxygen METabolism (COMET) tool that measures *in vivo* mitochondrial oxygen tension. The study by Costerus et al. showed feasibility in its use in neonates and this may be another promising point-of-care test or bedside patient monitoring system to be introduced in the NICU. In hereditary neurological conditions that are largely incurable, Wong et al. reviewed the role of gene-editing models using the Clustered Regularly Interspaced Short Palindromic Repeat (CRISPR) approach, providing a glimmer of hope in therapy in spite of the several drawbacks that need to be overcome such as vulnerability of neuronal cells and less efficient CRISPR delivery into the brain.

## Developmental Origins and Sex Differences in Health And Diseases

Finally, the developmental origins of health and diseases (DOHaD), a concept that was first proposed by Barker two decades ago, suggests that environmental exposure during the critical period in early life may program the later health in adulthood (18). In the review by Chin and Pang, rodent models of exposure to soy isoflavones during the neonatal period increased bone mineral density in adulthood of especially the female sex. This begs the question if the female or male sex exerts different influences on DOHaD. Sex differences in health outcomes are increasingly being detected with *post-hoc* analyses of clinical studies adjusting for sex as an independent factor (19–21). The gender disparity need to be actively pursued in future research to select sex as an independent and important variable. In the article by Bæk et al. their preterm piglet model receiving a standardized rearing protocol showed increased early mortality among males, which also showed slower growth. The group proposed to use this model for the study of differences in outcomes between the male and female sex with regards to neonatal care and responses to treatment in the future.

## Author Contributions

All authors listed have made a substantial, direct and intellectual contribution to the work, and approved it for publication.

## Conflict of Interest

The authors declare that the research was conducted in the absence of any commercial or financial relationships that could be construed as a potential conflict of interest.
